# Anterior Segment Neovascularization in Diabetic Retinopathy: A Masquerade

**DOI:** 10.1371/journal.pone.0123627

**Published:** 2015-06-01

**Authors:** Yong Cheng, Jinfeng Qu, Yi Chen, Mingwei Zhao, Xiaoxin Li

**Affiliations:** 1 Department of Ophthalmology, People’s Hospital, Peking University, Beijing, China; 2 Key Laboratory of Vision Loss and Restoration, Ministry of Education, Beijing, China; 3 Beijing Key Laboratory of Diagnosis and Therapy of Retinal and Choroid Diseases, Beijing, China; Medical University Innsbruck, AUSTRIA

## Abstract

**Purpose:**

Anterior segment neovascularization (ASNV) could be a masquerade for ocular ischemic syndrome (OIS) in diabetic patients which misleads diagnosis and treatment. The purpose of our study is to find the relationship between blood flow velocity in carotid siphon and the development of ASNV in diabetic.

**Methods:**

We reviewed 34 eyes of 17 diabetic patients who had Transcranial Color Doppler (TCD) examination with unilateral ASNV. The circulatory parameters of both eyes of each patient were compared and analyzed. In addition, 9 patients with more than 50% stenosis of extracranial internal carotid artery (ICA) and low velocity flow through TCD had been treated by carotid revascularization surgery.

**Results:**

The maximal velocity in systole (Vmax) of carotid siphon (SCA) was lower in the eyes with ASNV than in the eyes without ASNV (P<0.05). ASNV of all the 9 patients regressed totally and BCVA improved significantly (*P*<0.05). Stenosis of ICA and arm-retina time (ART) decreased significantly (*P*<0.01) and SCA and **ophthalmic artery (OA)** increased significantly (*P*<0.01).

**Conclusions:**

Our study showed ASNV could be a masquerade for OIS in patients with diabetic retinopathy. The decreased blood flow velocity in carotid siphon is related to the development of ASNV. Circulatory parameters screening of SCA by TCD is important to help us to evaluate the blood flow in SCA, the possibility of development of ASNV, and the prognosis of the patient. Interference such as carotid endarterectomy (CEA) or carotid artery stenting (CAS) can be performed if necessary to improve the blood flow in SCA and make ASNV regression.

## Introduction

Persons with diabetes are at risk of developing diabetic retinopathy (DR) and having it progress to proliferative DR (PDR), macular edema (ME) and anterior segment neovascularization (ASNV) [1]. ASNV includes angle neovascularization (ANV) and iris neovascularization (INV). It can lead to neovascular glaucoma (NVG) which is a severely blinding, intractable disease. The retinal and choroidal ischemia/hypoxia are considered the exciting factor for the development of ASNV. The presence of ASNV in diabetic always implies underlying retinal detachment with anterior proliferative vitreoretinopathy (PVR), severe ischemia retinopathy without enough coagulation, inflammation or ocular ischemic syndrome (OIS) [2, 3].

Ocular ischemic syndrome is a rare condition, which is caused by ocular hypoperfusion due to stenosis or occlusion of the common or internal carotid arteries. It is a frustrating condition for the ophthalmologist. The visual prognosis and treatment outcomes are poor. There is no well-established treatment. Moreover, it may be overlooked or misdiagnosed, primarily because of its diverse and sometimes subtle presentation[4, 5]. In the study of Mizener on ocular ischemic syndrome, the prevalence of diabetes mellitus in these patients was much higher than in the comparable general population[5]. As a ophthalmologist, we usually focus on retinal detachment with PVR, severe ischemia retinopathy without enough coagulation, however, ignore OIS. Therefore, ASNV could be a masquerade in diabetic which misleads diagnosis and treatment. To prevent and reduce the extent of visual loss caused by ASNV, the first priority should be looking for predictable risk factors of ASNV and trying to interfere its development by appropriate management of the causative diseases.

Ophthalmic Color Doppler imaging (CDI) is most commonly used to investigate circulatory parameters in the retrobulbar blood vessels and to evaluate retinal and choroidal perfusion. But the reproducibility of the measurement of the blood flow velocity in the retrobulbar vessels using CDI varies depending on the vessel imaged[6–10], the equipment used[6–10], the pressure that the examiner applied to the globe[6, 7], patient posture[11], eye movement and where the Doppler gate is positioned[6, 9, 10]. Misinterpretation of the Doppler spectrum by the software will lead to false results whenever the Doppler signal is noisy[6]. The coefficient of variation of blood flow velocity measured by CDI was reported to be 5–39%[7]. All these limitations make it difficult to analyze and explain the conflicting results that CDI give to us.

Carotid Doppler can help us evaluate blood flow in the carotid but it can only detect stenosis of the carotid artery in the neck but not above or below that. The blood flow in the extracranial carotid and their relationship with ASNV had been well studied but the blood flow in the carotid siphon was usually overlooked[11].

Transcranial Color Doppler (TCD) imaging of the carotid siphon can provide information about the blood flow in carotid siphon where in most cases the ophthalmic artery arising from[12].

The purpose of our study is to find the relationship between blood flow velocity in carotid siphon and the development of ASNV in diabetic.

## Methods

### Ethical statement

The study protocol was approved by the Ethics Committee for Human Research of Peking University People’s Hospital (No. 200903007) and adhered to the tenets of the Declaration of Helsinki. The study is in accordance with HIPAA regulation. Written informed consent was obtained from all study subjects. We explained risk and benefits on the consent form. Participants were voluntary, and individuals could withdraw from the trial at any time.

### Procedure

We reviewed the charts of the diabetic patients who had TCD examination with unilateral ASNV from 2009 through 2013. The circulatory parameters including internal diameter (ID), intima-media thickness (IMT), maximal velocity in systole (Vmax), minimal velocity in late diastole (Vmin), mean velocity (Vtamx), pulsative index (PI) and resistance index (RI) in the common carotid artery (CCA), extracranial internal carotid artery (ICA), carotid siphon (SCA) and ophthalmic artery (OA) of the both eye of each patients were compared and analyzed. All the circulatory parameters were measured by one experienced examiner.

34 eyes of 17 diabetic patients with unilateral ASNV were included in our study totally (Table 1). The mean age± standard deviation[SD] of the patients was 60.88±6.36years. The eyes were diagnosed as PDR without retinal detachment or PVR and with panretinal photocoagulation and without nonperfusion area through fluorescein angiography (FA). In addition, 9 patients with more than 50% stenosis of extracranial internal carotid artery (ICA) and low velocity flow through TCD had been examined by digital subtraction angiography(DSA), and then treated by carotid artery stenting (CAS) or carotid endarterectomy (CEA). All patients underwent a complete ophthalmic examination at each visit, including assessment of BCVA using standardized refraction with a Snellen chart and converted to logarithm of the minimum angle of resolution (logMAR) units for analysis, biomicroscopic evaluation, intraocular-pressure measurement with an applanation tonometer, and funduscopic examination. FA images were taken at pretreatment examination and at every visit. Patients were then followed monthly until six months by clinical, TCD and FA examinations.

**Table 1:** Characteristics of the study population.

Case Number	Tender	Age	DM	Hypertension	Cardiovascular Disease	Hyperlipidemia
**1**	M	78	DR	**+**	**-**	**-**
**2**	F	62	PDR	**+**	**-**	**+**
**3**	F	56	PDR	**-**	**-**	**-**
**4**	F	64	PDR	**-**	**+**	**+**
**5**	F	67	PDR	**+**	**+**	**+**
**6**	F	59	PDR	**-**	**-**	**-**
**7**	M	64	PDR	**+**	**-**	**-**
**8**	M	61	PDR	**+**	**+**	**+**
**9**	M	60	DR	**+**	**-**	**-**
**10**	M	49	PDR	**-**	**-**	**-**
**11**	F	59	PDR	**+**	**-**	**+**
**12**	M	56	PDR	**+**	**-**	**-**
**13**	F	57	PDR	**+**	**-**	**-**
**14**	M	67	PDR	**+**	**-**	**-**
**15**	M	63	PDR	**+**	**-**	**+**
**16**	F	55	PDR	**+**	**-**	**-**
**17**	F	58	PDR	**+**	**-**	**-**

**Abbreviations:** M, male; F, female; DR, diabetic retinopathy; PDR, proliferative diabetic retinopathy;

### Statistical analysis

Statistics were analyzed with SPSS Statistics 19.0. Statistical analyses were performed with Student’s t test. Paired-t test was used to compare the circulatory parameters between both eyes of the same patients and independent-Sample t test was used to compare the circulatory parameters between pre- and posttreatment values. Values of P<0.05 were considered statistically significant.

## Results

### The relationship of blood flow velocity in carotid siphon and the development of ASNV in all subjects

Paired-t test showed there was no significant difference in circulatory parameters of CCA, extracranial ICA, and OA between eyes with and without ASNV in the same patient (Table 2). The Vmax of SCA was lower in the eyes with ASNV than in the eyes without ASNV (79.65±21.44 cm/s vs. 83.41±23.59 cm/s, P<0.05) (Table 2).

**Table 2:** Comparison of circulatory parameters in CCA, extracranial ICA, SCA and OA between eyes with and without ASNV.

	Circulatory Parameters	ASNV(+)		ASNV(-)		P value
		n	mean	n	mean	
**CCA**	ID (mm)	17	6.66±1.22	17	6.66±1.12	0.966
	IMT (mm)	17	0.89±0.22	17	0.88±0.19	0.549
	Vmax (m/s)	17	0.58±0.18	17	0.64±0.19	0.211
	Vmin(m/s)	17	0.14±0.06	17	0.15±0.06	0.280
	Vtamx(m/s)	17	0.26±0.09	17	0.28±0.09	0.505
	PI	17	1.66±0.36	17	1.72±0.34	0.333
	RI	17	0.76±0.07	17	0.76±0.06	0.610
**Extracranial ICA**	ID (mm)	17	4.29±0.97	17	4.38±0.90	0.489
	IMT (mm)	17	0.84±0.14	17	0.87±0.19	0.266
	Vmax (m/s)	17	0.60±0.17	17	0.59±0.19	0.896
	Vmin (m/s)	17	0.23±0.08	17	0.22±0.08	0.743
	Vtamx (m/s)	17	0.35±0.11	17	0.34±0.13	0.768
	17	17	1.04±0.24	17	1.04±0.24	0.992
	PI	17	0.61±0.07	17	0.61±0.09	0.975
**SCA**	Vmax (cm/s)	17	79.65±21.44	17	83.41±23.59	0.035*
**OA**	Vmax (cm/s)	17	34.94±12.52	17	37.06±8.81	0.198

**Abbreviations:** CCA, Common carotid artery; ICA, Internal carotid artery; SCA, Carotid siphon; OA, Ophthalmic artery; ID, internal diameter; IMT, intima-media thickness; Vmax, maximal velocity in systole; Vmin, minimal velocity in late diastole; Vtamx, mean velocity; PI, pulsative index; RI, resistance index.

### Comparison of pre- and posttreatment values in the nine treated patients

Table 3 shows the comparison of pretreatment and posttreatment in sixth months BCVA, arm-retina time(ART), stenosis of ICA, SCA, OA in nine treated patients. ASNV of all the 9 patients regressed totally and BCVA improved significantly (*P*<0.05). Stenosis of ICA and ART decreased significantly (*P*<0.01) and SCA and OA increased significantly (*P*<0.01).

**Table 3:** Comparison of pre- and posttreatment values in the nine treated patients.

Case Number	Operation	Pre-operation						Post-operation					
		ASNV	BCVA (LogMAR)	ART(s)	stenosis of ICA	SCA(cm/s)	OA(cm/s)	ASNV	BCVA (LogMAR)	ART(s)	stenosis of ICA	SCA(cm/s)	OA(cm/s)
1	CEA	(+)	2.00	26.0	90%	81	19	(-)	2.00	19.3	30%	113	22
3	CEA	(+)	1.70	20.3	70%	78	30	(-)	0.70	17.8	20%	95	37
4	CEA	(+)	1.70	25.1	80%	73	21	(-)	0.80	19.1	30%	103	31
7	CEA	(+)	2.00	29	70%	64	30	(-)	2.00	18.3	20%	80	32
8	CEA	(+)	2.00	24.2	80%	68	18	(-)	1.70	17.3	30%	145	24
10	CEA	(+)	1.70	23.2	50%	130	18	(-)	0.40	16.7	10%	182	26
12	CEA	(+)	2.00	19.3	80%	87	24	(-)	1.40	16.6	30%	146	33
14	CAS	(+)	0.52	18.1	60%	78	29	(-)	0.52	16.0	30%	101	38
17	CEA	(+)	2.00	21.3	70%	72	24	(-)	0.49	18.1	20%	107	41

**Abbreviations:** ASNV, Anterior segment neovascularization; VA, Visual acuity; HM, Hand movement; FC, Finger counting; ART, Arm-retina time in fluorescein angiography. ICA, Internal carotid artery; SCA, Carotid siphon; OA, Ophthalmic artery; CEA, Carotid endarterectomy; CAS, Carotid artery stenting.

**Fig 1:**
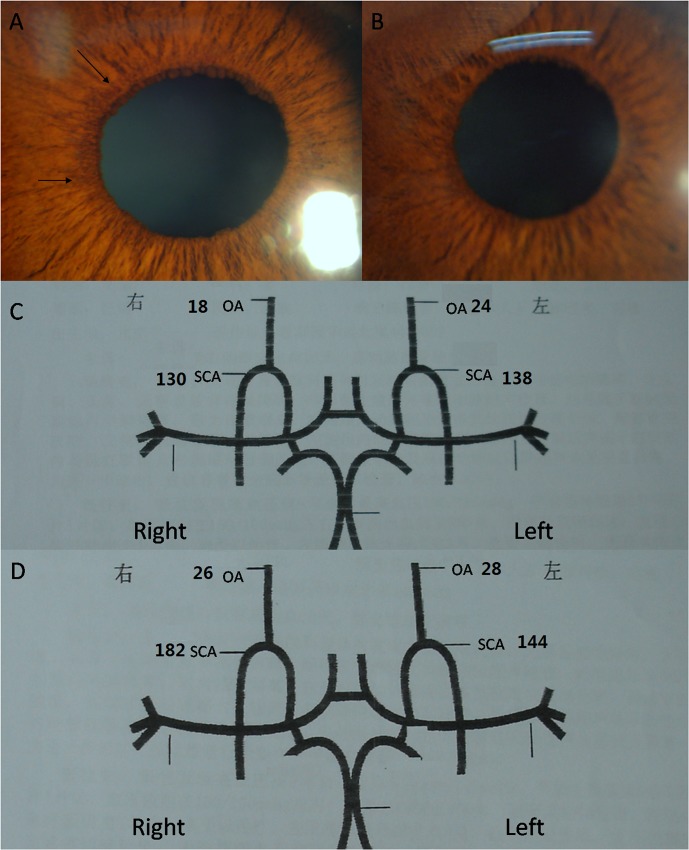
ASNV disappeared with Vmax in right SCA and OA improved after CEA in patient 1. A, INV can be seen at the pupil margin from 9 to 11 o’clock in the right eye before CEA. B, INV in the right eye disappeared at 10 days after CEA. C, TCD examination showed lower Vmax in right SCA and OA comparing to the left side before CEA in the right ICA. D, TCD examination showed improved Vmax in right SCA and OA comparing to the left side at 10 days after CEA in the right ICA.

Here we want to show a representative case of patient 10 who underwent carotid endarterectomy (CEA). He was a 49 years old Chinese male. He had had type 2 diabetes and hypertension for 3 years. When he presented to us, his BCVA in LogMAR was 1.70 in the right eye and 1 in the left eye. His intraocular pressure was normal in both eyes. Slit lamp examination found new vessels at the pupil margin from 9 to 11 o’clock in the right eye but no ASNV in the left eye. B ultrasound showed vitreous hemorrhage in both eyes without retinal detachment. He was diagnosed as binocular PDR with vitreous hemorrhage and INV in the right eye. CDI examination of his CCA and extracranial ICA showed 50% stenosis of the right extracranial ICA which was confirmed by carotid arteriography. TCD examination showed lower Vmax in right SCA and OA comparing to the left side. Circulatory parameters of extracranial ICA, SCA and OA were showed in Table 3. He underwent CEA of right ICA because of 50% stenosis of the right extracranial ICA. 6 months after CEA, the INV in his right eye regressed totally (Fig 1A and 1B).TCD showed his Vmax in right SCA improved from 130 cm/s to 182 cm/s, Vmax in right OA improved from 18 cm/s to 26 cm/s (Fig 1C and 1D).

## Discussion

The vascular complications in diabetic patients comprise large vessel disease, macroangiopathy, and also diabetic microangiopathy that is considered to be specific to diabetes. Macroangiopathy is characterized by ischemic heart disease, cerebrovascular disease and peripheral vascular disease[13]. Carotid occlusive disease, one of the cerebrovascular diseases, is a common cause of stroke and presents with several ocular manifestations, collectively referred to as the ocular ischemic syndrome (OIS)[14, 15]. When OIS is associated with diabetes mellitus, the ocular manifestations of both diseases may be superimposed, which may lead to some confusion.

Previous studies reported an association of carotid occlusive disease and diabetes in the development of ASNV [16]. When ASNV was examined, as a ophthalmologist, we focused on retinal detachment with PVR, severe ischemia retinopathy without enough coagulation, however, ignored OIS. Therefore, ASNV could be a masquerade in diabetic which misleads diagnosis and treatment. The development of ASNV and subsequent NVG is usually difficult to manage and often resulting in disastrous visual loss. Moreover, carotid artery occlusion often progresses without symptoms, and when the patient notices an ocular disorder

and visits a clinic, the condition is often at an advanced stage of ocular ischemia, in which neovascular glaucoma has developed with severe internal carotid artery stenosis[17]. To prevent or reduce the visual loss caused by ASNV, the first priority should be looking for predictable risk factors of ASNV and try to interfere its development by appropriate management of the causative diseases.

It is not uncommon in clinical practice to find that carotid artery disease is ruled out as the cause of ocular ischemia based on findings of absence of occlusion or severe stenosis of the internal carotid artery on carotid Doppler. However, carotid Doppler evaluates only the artery in the neck and not the siphon part of ICA where it may be stenosed. In our study, we not only use carotid Doppler to compare the circulatory parameters of CCA and extracranial ICA, but also use TCD to compare the circulatory parameters of SCA and OA between eyes with and without ASNV in the same patient. The variation caused by the factors that may affect the ocular circulation including the type of diabetes, age, metabolic control, and systemic therapy (anti-hypertensives, dyslipidemia agents) were all ruled out[10]. The results showed there was no significant difference in circulatory parameters of CCA, extracranial ICA, and OA between eyes with and without ASNV in the same patient, but the Vmax of SCA was significantly lower in the eyes with ASNV than in the eyes without ASNV. The decreased blood flow velocity in SCA can be caused by the stenosis of CCA or any part of ICA before siphon. Screening of SCA by using TCD provides us the information about blood flow changes caused not only by stenosis CCA or extracranial ICA but also by stenosis of intracranial ICA before or at siphon. This finding shows the importance of circulatory parameters screening of SCA by using TCD and remind us to be precautious about the disturbance of the circulatory changes in SCA which may be related to the development of ASNV.

Winfried Goebe compared the blood velocity measurements of OA, PCA, and CRA in the right and the left eye of the same patient in PDR group by using ophthalmic CDI and found that there is a highly significant correlation between the two eyes[6]. In our study, we didn’t find difference in Vmax of OA in the right and the left eye of the same PDR patient either. OA provides the retinal artery flow. We also thought that ophthalmic artery Vmax should show the statistically significant difference between the ASNV (+) group and ASNV (-) group. However, the result did not show that. We thought it was because the sample was small. In addition, we found the Vmax of SCA was lower in the eyes with more severe PDR than the other eye in the same PDR patient. This difference in the circulatory parameters of SCA gave us a better explanation to the difference of PDR severity in the same patient which is commonly seen in clinical practice. We presume that the decreased blood flow velocity in carotid siphon causes more severe retinal and choroidal ischemia/hypoxia.

Carotid revascularization surgery, such as carotid endarterectomy (CEA) and carotid artery stenting (CAS), may also restore the cerebral perfusion pressure and improve the intracranial vascular hemodynamics including ocular circulation[18–22]. Therefore, carotid artery revascularization surgery reduces the risk of stroke in symptomatic and asymptomatic patients[23, 24]. Despite this well-established benefit, there have been few reports concerning the effect of carotid revascularization surgery on ocular circulation. In this study, we found the significant improvements of the average SCA and OA peak systolic flow velocities and the correction of stenosis of ICA and ART were sustained throughout the six months of follow-up. For the patients with chronic ocular ischemic syndrome due to disturbed ocular circulation, it is vital to correct the ocular circulation to prevent and improve ocular ischemia[25]. In this series, ASNV of all the 9 patients regressed totally and BCVA improved significantly. Moreover, carotid artery revascularization reduces the risk of stroke in symptomatic and asymptomatic patients[24]. Improvements in surgical and endovascular techniques have reduced the incidence of ischemic stroke following CEA and CAS, respectively. ASNV develops especially in patients with poor collateral circulation between the internal and external carotid arterial systems or between the two ICAs. Those with well-developed collateral circulation may not develop ASNV even with total occlusion of the ICA. On the contrary, in those with poor collaterals a stenosis of less than 50% of the ICA may be sufficient for the ASNV to develop[2, 15, 26]. In our study, patient 10 underwent CEA because of the presence of ASNV and 50% stenosis of ICA. He showed regression of ASNV and the improvement of Vmax in SCA after the only treatment of CEA. This finding on the other hand proved that decreased blood flow velocity in carotid siphon is a highly related to the development of ASNV.

In this study, decreased blood flow velocity in carotid siphon is relatedto the development of ASNV. However, it remains unknown whether there is a close correlation between a reduction in blood flow velocity and volumetric flow[6]. The present models of the retinal circulation used to calculate volumetric flow from diameter and velocity measurements assume a constant cylindrical vessel geometry and velocity profile. Yet, changes in vessel shape in pathologic conditions such as diabetic retinopathy must be taken into account. Grunwald and coworkers have presented data from the evaluation of fundus photographs confirming a statistically significant enlargement of venous and arterial vessel diameters in patients with diabetes compared to normal controls[27]. Vasodilation, however, leads to decreased blood flow velocity even without any change in volumetric flow. Therefore, a decreased flow velocity might still reflect unchanged volumetric flow when marked vasodilation is present.

In summary, our study showed the decreased blood flow velocity in carotid siphon is related to development of ASNV. Circulatory parameters screening of SCA by using TCD is important to help us to evaluate the blood flow in SCA, the risk of development of ASNV, and the prognosis of the patient. Interference such as CEA can be performed if necessary to improve the blood flow in SCA and make ASNV regression. It is important to recognize the underlying carotid occlusive disease in diabetic patients because of the poor visual prognosis and high mortality. We should manage not only the diabetic retinopathy, but also the carotid atherosclerosis to preserve good visual function in diabetic patients.
